# FedETC: Encrypted traffic classification based on federated learning

**DOI:** 10.1016/j.heliyon.2024.e35962

**Published:** 2024-08-11

**Authors:** Zhiping Jin, Ke Duan, Changhui Chen, Meirong He, Shan Jiang, Hanxiao Xue

**Affiliations:** a School of Information Engineering, Zhongshan Polytechnic, Zhongshan, China; b Guangzhou Panyu Polytechnic, Guangzhou, China; c Institute of Artificial Intelligence, Guangzhou University, Guangzhou, China; d School of Information Technology and Engineering, Guangzhou College of Commerce, Guangzhou, China

**Keywords:** Network traffic classification, Federated learning, Encrypted traffic

## Abstract

The current popular traffic classification methods based on feature engineering and machine learning are difficult to obtain suitable traffic feature sets for multiple traffic classification tasks. Besides, data privacy policies prohibit network operators from collecting and sharing traffic data that might compromise user privacy. To address these challenges, we propose FedETC, a federated learning framework that allows multiple participants to learn global traffic classifiers, while keeping locally encrypted traffic invisible to other participants. In addition, FedETC adopts one-dimensional convolutional neural network as the base model, which avoids manual traffic feature design. In the experiments, we evaluate the FedETC framework for the tasks of both application identification and traffic characterization in a publicly available real-world dataset. The results show that FedETC can achieve promising accuracy rates that are close to centralized learning schemes.

## 1 Introduction

The classification and identification of network traffic play a significant role in network operation management and cyberspace security [1], [2], [3]. For instance, identifying the type of applications in a network enables fine-grained management of critical bandwidth resources. It also serves as an effective and essential building block of network protection for detecting unknown attack behaviors, preventing network intrusions, and facilitating network situational awareness [4], [5].

It is worth noting that with the evolution and development of encryption protocols such as Transport Layer Security (TLS), the era of traffic encryption has come. Network traffic, as the core carrier of network data, can hide user behavior and protect user privacy by using encryption technology. Traditional network traffic classification approaches like deep packet inspection rely on matching protocol signatures in the application layer data, which will face a huge challenge to process encrypted traffic data [6], [7]. In contrast, machine learning based approaches can effectively handle encrypted traffic [8].

Generally, encrypted traffic classification based on classic machine learning require researchers to design and define a set of traffic feature in advance and then train a classification model using machine learning algorithms such as Naive Bayes, support vector machine, decision trees and so on. Nonetheless, due to the complexity of network applications and the prominent difference in traffic components, the manually defined feature sets will significantly affect the performance of traffic classifiers. For example, network operators have a wide variety of traffic types, and thus need to have experienced network experts to manually construct a representative set of features, which incurs expensive labor costs [9]. In addition, manual feature engineering cannot adapt to the current surge of network applications with significantly different behaviors and concept drifts. Deep learning [10], on the other hand, differs from classic machine learning algorithms in that it can accomplish both feature learning and traffic classification without requiring the manual efforts of network experts. It not only reduces labor costs linked to feature design but also facilitates the discovery of deep traffic patterns [11]. In recent years, a series of studies have adopted deep learning for classifying encrypted network traffic and reported promising results [12], [13], [14].

Despite the promising results of machine learning and deep learning approaches for network traffic classification, most existing studies focus on centralized learning schemes and rely on the quality of training data. In practice, collecting a large, representative and diverse traffic data set is difficult and subject to security and privacy concerns [15]. While centralized learning schemes can aggregate traffic data from many network domains to improve data quality and model performance, the participating domains do not have full control over their data, which can lead to data leakage. For example, although the payload of SSL/TLS packet is invisible, a lot of side information such as the length of packets, the interval between packets, the dependency relationship between packets, and the transmission relationship between packets can be disclosed. More importantly, due to the frequent occurrence of user privacy leakage caused by centralized training, many countries around the world have introduced data privacy protection laws and policies.

In this paper, we propose the FedETC (Federated Encrypted Traffic Classification) framework, which allows multiple autonomous network domains to collaborate with each other on training a universal end-to-end encrypted traffic classification model without disclosing their data to other participants and the central server. The goal is to achieve classification accuracy and data privacy at the same time. In FedETC, the traffic sessions are transformed and represented in normalized byte sequences with fixed length. The sequences are provided as input to train the one-dimensional Convolutional Neural Network (1D-CNN) model. The parameters of the local models are exchanged with the central server and merged into a global model using the FedAvg algorithm.

For the purpose of evaluation, we conduct extensive experiments using a publicly available encrypted traffic dataset. In the experiments, we perform two types of traffic classification tasks (i.e., application identification and traffic characterization), and also consider two scenarios with different class and data distributions across the participating network domains. The results show that the proposed federated learning scheme can achieve accuracy rates that are close to that of the centralized learning scheme. The difference in overall accuracy can be as low as 0.8%.

The contributions of this work are summarized as follows:

• We propose a federated learning scheme for encrypted traffic classification, which is based on 1D-CNN model and FedAvg algorithm.
• We compare the proposed scheme with centralized and other federated learning approaches using a publicly available real-world encrypted traffic dataset.
• We explore the impact of different distributions of traffic class and data across the participants in the proposed federated learning.

The rest of this paper is organized as follows. Section 2 provides a brief review of the related works. Section 3 introduces the FedETC framework and describes the components in detail. The experimental results and analysis are presented in Section 4. Finally, section 5 concludes the paper.

## 2 Related works

Traffic classification approaches based on machine learning often use a pre-defined feature set that consists of statistical traffic characteristics [16], [17], [18], [19], [20], [21]. For example, Riyad et al. [22] use more than 20 statistics data, such as the mean forward arrival interval and the minimum forward packet length, as the input of the machine learning algorithm, and identified 14 traffic attributes for Secure Shell (SSH) traffic classification. Moore et al. [23] identify near 250 features that are useful for classifying flow records. However, more features result in higher computational cost and thus limit the application in real-time traffic classification. Therefore, feature selection or feature reduction techniques are often adopted for dimension reduction and redundancy removal.

To avoid the disadvantages of manual feature design, researchers begin to adopt the deep learning models that integrate feature learning. For example, Zhou et al. [24] adopt the minimum-maximum normalization method to process network flow data and map it into gray-scale images as the input data of the convolutional neural network to achieve feature learning. Rui et al. [25] provide a byte segment neural network for traffic classification, in which the payload fragments are put into the attention encoder to automatically obtain feature representation vectors, and then the softmax classifier is used for classification.

In recent years, encrypted traffic classification has attracted more and more research attention due to the widespread use of traffic encryption. For example, Wang et al. [12] convert encrypted traffic into gray-scale images as the input of a 1D-CNN and perform classification tasks of different granularity. Lotfollahi et al. [13] use stacked autoencoder and Convolution Neural Network (CNN) to automatically extract the effective load characteristics of encrypted packets to identify traffic. Aceto et al. [14] propose the Distiller classifier, which adopts a multi-modal multitask deep learning approach for encrypted traffic classification.

Other than the traditional centralized learning scheme adopted in the above-mentioned approaches, federated learning has also been applied in the field of network traffic classification. For example, Mun et al. [15] propose a federated traffic classification scheme to identify traffic by associating locally encrypted data across various traffic transmission devices.

## 3 Method

This section first introduces the proposed framework, then describes the data preprocessing process, classification model, and federated learning algorithms in detail.

### 3.1 Framework

**Figure 1:**
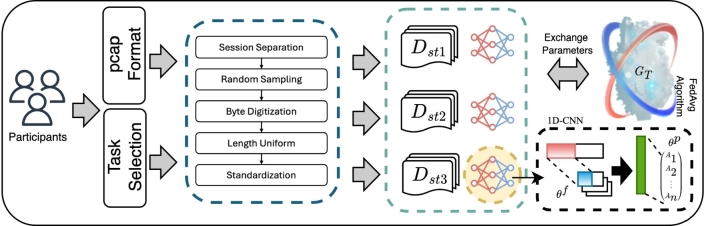
The FedETC framework.

The proposed FedETC framework for encrypted traffic classification is illustrated in Fig. 1. The figure shows the main process how the participating domains implement the federated learning scheme to collaboratively train the 1D-CNN model without disclosing local training data.

In this work, we are interested in different encrypted traffic classification tasks including application identification and traffic characterization. As listed in Table 1, application identification (APPI) involves identifying specific applications such as Facebook, Gmail, Skype, Netflix, Spotify, YouTube and so on, while traffic characterization involves breaking the traffic down to categories such as chat, email, file transfer, streaming and so on, with and without virtual private network (VPN) tunneling and encryption.

**Table 1:** Encrypted traffic classification tasks.

Task	Classes
APPI: Application Identification	Aim Chat, Email, Facebook, FTPS, Gmail, Hangouts, ICQ, Netflix, SCP, SFTP, Skype, Spotify, Tor, Torrent, Vimeo, Voipbuster, Youtube.
TC: Traffic Characterization	Chat, Email, File Transfer, Streaming, Torrent, VoIP, VPN Chat, VPN Email, VPN File Transfer, VPN Streaming, VPN Torrent, VPN VoIP.

After selecting the interested task, the network operators of the participating domains can collect and label local traffic data within their domain for the purpose of training. The collected traffic data is stored in pcap format. Several data preprocessing steps are involved to derive the training set, which is then fed into the local 1D-CNN model for training.

A central server is coordinating the participating nodes to build a global model. It is done by communicating with the participants to exchange model parameters and running the FedAvg federated learning algorithm to merge the models. Please note that the data is processed and used for training locally within each participating domain, only the model parameters will be exchanged beyond the domain boundaries.

### 3.2 Data preprocessing

**Figure 2:**
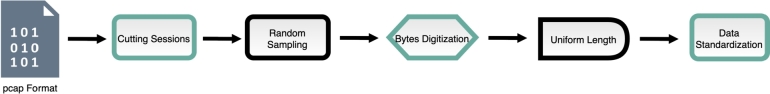
Data preprocessing operations.

The data preprocessing consists of traffic cleaning, session cutting, bytes digitization, uniform length, and data standardization, as shown in Fig. 2.

Typically, a traffic trace collected from a network consists of many sessions. Each session *S* can be identified by and represented as in Equation (1).

(1) $$S=\{f_{i}|i:(sip,dip,sport,dport,pro)\},$$

where *i* indicates the 5-tuple (source IP address, source port number, destination IP address, destination port number, and transport protocol) that occurs during session establishment, and $f_{i}$ indicates a one-way flow.

The collected traffic data is stored in the binary form as in the pcap files, where each session *S* can be represented by a vector of bytes *v* as in Equation (2).

(2) $$v=\{b_{1},b_{2},...,b_{i},...\},$$

where $b_{i}$ represents the *ith* byte of the data in the session. The structural relationships in the byte sequence can be destroyed if the sequence is reorganized. Therefore, we adopt the form of one-dimensional byte vector for data representation.

We then convert the original byte vector *v* of the encrypted traffic into a sequence of integers *d* as in Equation (3).

(3) $$d=\{d_{1},d_{2},...,d_{i},...,d_{n}\},$$

where $d_{i}$ represents the value of $b_{i}$ ranging from 0 to 255. Vector *d* is set to have a fixed length *n*. For example, we set *n* to 700 in our experiments. The sequences longer than *n* bytes are truncated and the sequences shorter than *n* bytes are padded with zeros.

Finally, we normalize the integer vector *d* as in Equation (4).

(4) $$D_{st}=\{D_{1},D_{2},...,D_{i}...,D_{n}\},$$

where $D_{i}=d_{i}/255\in [0,1]$. The normalized fixed length vector are aggregated to form the training data set $D_{st}$, which is used as the input of the local 1D-CNN model.

### 3.3 Classification model

In this work, we adopt 1D-CNN as the base classification model. 1D-CNN has been adopted in several previous studies in traffic classification and showed promising results [26], [27], [28]. It is considered to be effective on dealing with sequential data.

Table 2 shows the network structure and parameters of the 1D-CNN model designed in our work. The model consists of two main parts: a feature extractor and a predictor. The feature extractor consists of multiple convolutional layers and pooling layers, while the predictor consists of multiple fully connected layers. The normalized data of 1*700 dimension *D* (n=700) is fed to the feature extractor, then transformed into a 1*16400 dimension vector, and finally fed to the predictor to output the prediction label.

**Table 2:** Parameters of 1D-CNN model (*n* = 700).

Layer	Operation	Input	Filter	Stride	Output
1	Conv+ReLU	700*1	4	2	349*100
2	MaxPool	349*100	3	1	347*100
3	Conv+ReLU	347*100	3	2	173*200
4	MaxPool	173*200	3	1	171*200
5	Conv+ReLU	171*200	3	2	85*300
6	MaxPool	85*300	3	1	83*300
7	Conv+ReLU	83*300	3	2	41*400
8	Linear	41*400	-	-	7600
9	Linear	7600	-	-	3800
10	Linear	3800	-	-	1800
11	Linear	1800	-	-	600
12	Linear	600	-	-	180
13	Linear	180	-	-	17

The model includes 4 convolution layers, 3 pooling layers, and 6 full connection layers. Suppose that a single-channel traffic data $D_{st}$ of 1⁎a dimension is input into a single convolution layer composed of 1⁎N neurons, and filtered by a convolution kernel with a size of 1⁎n and a sliding step of *s*, and the result $O^{Conv_{L}}$ in the output layer $Conv_{L}$ is calculated as in Equation (5).

(5) $$O_{ij}^{Conv_{L}}=\sum_{n=1}^{n}W_{m,j}^{0}{D_{st}}_{i+n-1,j}^{0}+B_{j}^{0},$$

where *i* represents feature index, *j* represents feature graph index, *W* represents weight and *B* represents bias. Signal $O_{ij}^{Conv_{L}}$ is converted to output signal $Out^{L}$ by applying a nonlinear activation function (e.g. ReLU), whose output signal $Out^{L}$ can be obtained as in Equation (6).

(6) $$Out_{ij}^{L}=f(O_{ij}^{Conv_{L}}),$$

where f(⋅) represents the nonlinear activation function. The same process applies to the signals at some pooling layers. These valuable signal $Out^{L}$ input to fully connected layers is used to output predictive labels. In summary, $D_{st}$ is first input into the feature extractor to obtain feature vectors with varying degrees of complexity, followed by input into the predictor to accomplish the classification.

### 3.4 Federated learning algorithm

Each network operator participant who choose to train a certain classification model have a small amount of encrypted traffic $D_{st}^{k}={\left\{(x_{i}^{k},y_{i}^{k})\right\}}_{i=1}^{N_{k}}$ that has been standardized by preprocessing. The server initializes a certain global model. Every time the server communicates with participants, each randomly selected participant k∈C⋅m receives the global model $G_{T}$ and parameters sent by the server and perform *E* rounds of supervised training locally, where *C* is the participant fraction that controls the amount of multi-participant parallelism. Parameters $\theta_{k}$ of the model $G_{T}$ includes parameters $\theta_{k}^{f}$ of feature extractor f(z|x) and parameters $\theta_{k}^{p}$ of predictor $p(y^{\prime }|z)$, so the loss risk assessment $L_{\theta_{k}}$ of the model can be calculated as in Equation (7).

(7) $$L_{\theta_{k}}=\frac{1}{N_{k}}\sum_{i=1}^{N_{k}}loss(p(f(x_{i}^{k},\theta_{k}^{f}),\theta_{k}^{p}),y_{i}^{k}),$$

where loss(⋅) is cross-entropy loss function. The server uses FedAvg algorithm to average and update local model parameters of participants until the global model $G_{T}$ is stable and stored in the server as in Equation (8).

(8) $$\theta \leftarrow \frac{1}{m}\sum_{k=1}^{m}\theta_{k}$$

Our goal is to minimize the loss of models to participants and to learn a shared model without sharing local data [29] as in Equation (9).

(9) $$min_{\theta }\sum_{k=1}^{m}L_{\theta_{k}}.$$

## 4 Experiment

### 4.1 Dataset

**Figure 3:**
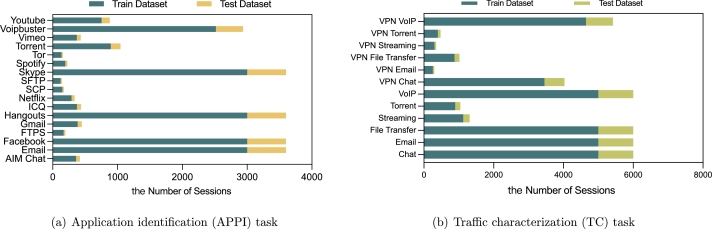
Number of per-class sessions in the dataset.

In order to evaluate the propose approach, we use the publicly available encrypted network traffic dataset ISCXVPN2016 [30]. The dataset is provided in pcap format and consists of representative real network traffic generated by Skype, Facebook, and other popular services, as showed in Table 1. For each type of traffic, both a regular session and a session over VPN are captured. The data is used for two classification tasks, that is, application identification (APPI) and traffic characterization (TC). Fig. 3 shows the number of per-class sessions obtained after data preprocessing, which are randomly divided into training data (85% per class) and testing data (15% per class).

In order to simulate the distributed data sets in the federated learning setting, we suppose there are 20 network domains to train a model jointly. Therefore, we need to divide the data into 20 parts. In particular, we consider two scenarios. One is the IID scenario, where the traffic is independent and identically distributed throughout all the network domains. The other is the non-IID scenario, in which each network domain could have different types and distributions of traffic. For example, domain A may have some FTPS traffic that is not seen in domain B.

**Figure 4:**
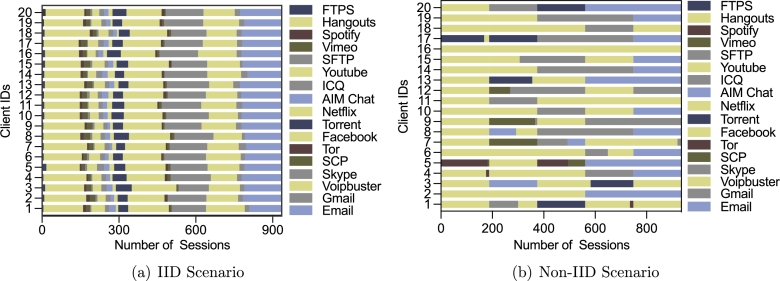
Number of samples in each participant for APPI task.

The divided dataset for the APPI task is shown in Fig. 4. In particular, Fig. 4(a) shows the IID scenario, in which we have the training data shuffled and divided per class label, resulting independent and identically distributed samples for each participant. Fig. 4(b) shows the Non-IID scenario, in which we first sort the training samples by class label and divide them into 100 fragments (with a size of 187 for each), and then randomly assign 5 fragments to each participant. It can be noticed that there are 5 dominating types of traffic (Voipbuster, Skype, Hangouts, Facebook, Email) in the APPI data set in Fig. 3(a). Therefore, some participants only have the samples from the 5 dominating classes, as seen in Fig. 4(b).

**Figure 5:**
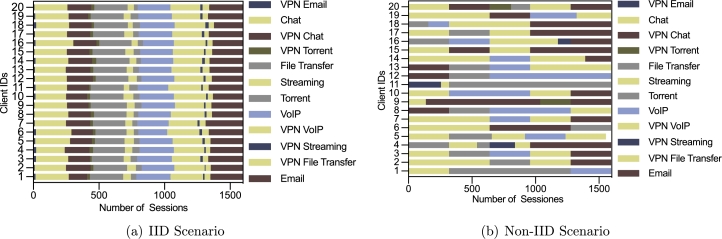
Number of samples each participant for TC task.

The dataset for the TC task is divided in the same way. Fig. 5(a) shows the case for the IID scenario, and Fig. 5(b) shows the case for Non-IID scenario.

### 4.2 Evaluation and validation metrics

In the case of multi-class classification with unbalanced class distribution, the overall accuracy cannot completely reflect the effectiveness of classifiers. Therefore, in addition to overall accuracy, the per-class metrics including precision (Pr), recall (Rc), and F1 score (F1) are included as evaluation metrics. Given a target class, precision reflects the correct predictions among the positive predictions to this class and recall reflects the correctly identified samples among all samples of this class. F1 value is a combination measure of precision and recall. The metrics are described mathematically as in Equation (10) to (13).

(10) $$Accuracy=\frac{TP+TN}{TP+TN+FP+FN},$$

(11) $$Pr=\frac{TP}{TP+FP},$$

(12) $$Rc=\frac{TP}{TP+FN},$$

(13) $$F1=\frac{2\cdot Pr\cdot Rc}{Pr+Rc},$$

where TP is the number of positive samples correctly classified as positive, FP is the number of negative samples that is wrongly classified as positive, TN is the number of negative samples correctly classified as negative, and FN is the number of positive samples that are wrongly classified as negative.

### 4.3 FedETC vs. centralized learning

We first conduct a set of experiments to compare the proposed FedETC framework to the centralized learning scheme. Centralized learning scheme uses all the training data as showed in Fig. 3. FedETC adopts the data partitions as showed in Fig. 4 and Fig. 5 for 20 participating client nodes.

**Figure 6:**
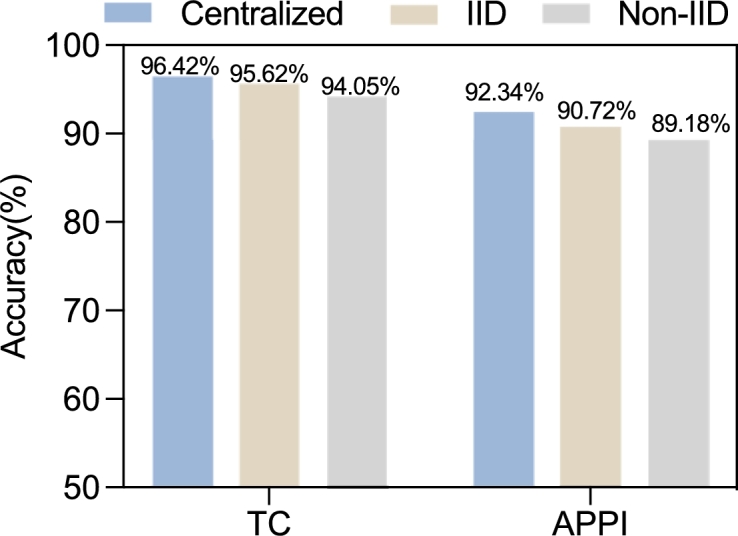
The accuracy results in different scenarios for TC task and APPI task.

As shown in Fig. 6, the accuracy of FedETC is comparable to that of centralized learning. For the TC task, the accuracy difference between the centralized method and FedETC is 0.8% in the IID scenario and 2.37% in the non-IID scenario. For the APPI task, the difference is 1.62% in the IID scenario and 3.16%in the non-IID scenario. When the accuracy rates of the two learning paradigms are comparable, the advantage of FedETC is that it allows different domains to cooperate while preserving data privacy.

**Figure 7:**
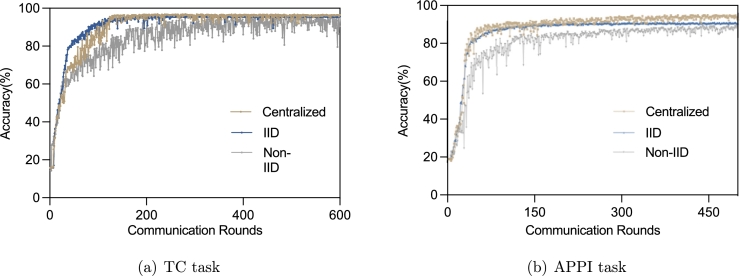
The accuracy results in different communication rounds.

Fig. 7(a) and Fig. 7(b) show the accuracy results in different communication rounds for the TC task and APPI task respectively. As can be seen, for both tasks the model convergence rate in the IID scenario is close to that in centralized learning. However, due to the influence of imbalanced and differently distributed samples in each participating domain, model convergence is much slower in the Non-IID scenario.

### 4.4 FedETC vs. FLIC

In this section, we compare FedETC with an existing federating traffic classification scheme called FLIC [15]. FLIC uses the same dataset to sort the first 1500 bytes of packet into 375*4 gray images (2-dimensional vector) and then employed the FedAvg algorithm to aggregate the convolution model to realize dynamic and static application recognition. The key difference between FedETC and FLIC lies in data preprocessing and convolution model structure. For the purpose of comparison, we perform the APPI task with all the data classes in Table 1 except Tor. The federating learning parameters are as C=0.1 and E=5.

Table 3 shows the F1 results in the APPI-IID task. For the classes with fewer samples, FedETC obtains much higher F1 scores than FLIC. For example, the F1 scores for AIM, Email and iCQ are 0.81, 0.98 and 0.84 for FedETC, in contrast to 0.59, 0.67 and 0.62 for FLIC. Besides, the average F1 scores of FLIC and FedETC are 0.87 and 0.90, respectively. The results indicate that FedETC performs better, especially for the classes with fewer samples.

**Table 3:** The F1 results for IID scenario in APPI task.

Application	FLIC	FedETC
AIM Chat	0.59	0.81
Email	0.67	0.98
Facebook	0.94	0.99
FTPS	0.98	0.9
Gmail	0.84	0.9
Hangouts	0.94	0.82
ICQ	0.62	0.84
Netflix	0.96	0.83
SCP	0.91	1
SFTP	0.86	0.81
Skype	0.89	0.78
Spotify	0.94	0.89
Torrent	0.94	0.98
Vimeo	0.97	0.85
Voipbuster	0.96	0.98
Youtube	0.95	0.97

**Average**	**0.87**	**0.90**

**Figure 8:**
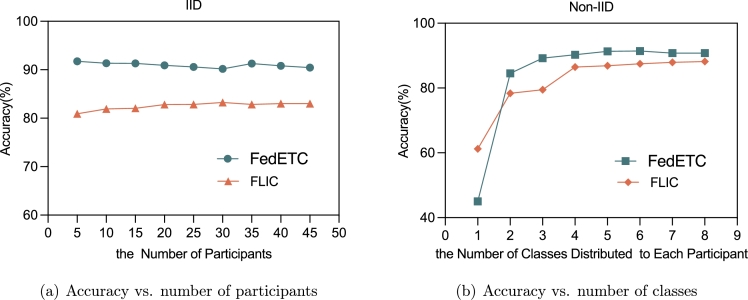
The accuracy results of FedETC and FLIC in comparison.

Fig. 8(a), demonstrates the different behavior of FedETC and FLIC by varying the number of participants from 5 to 45. As shown in the results, FedETC is more accurate than FLIC in the IID scenario. Besides, the trends show that both schemes maintain steady performance while increasing the number of participants, as long as their data is IID.

To evaluate the influence of Non-IID data in the APPI task, we explore the impact by increasing or decreasing the number of classes presented in each participant. As depicted in Fig. 8(b), in the rare case that only one class of traffic is presented in the dataset of each participant, FedETC obtains poorer accuracy than FLIC. However, when each participant has two or more classes of traffic in their data, FedETC can achieve better accuracy than FLIC.

## 5 Conclusion

In this work, we propose a federated learning approach for encrypted traffic classification. We use 1D-CNN model to learn from the encrypted traffic sessions presented in the form of normalized fixed-length byte sequences, which integrates the process of feature learning and prediction. The federated learning scheme allows autonomous network domains to perform collaborate model training without exposing local data. The proposed approach is evaluated based on a public dataset with comparison to the classic centralized learning scheme and an existing federated learning approach FLIC. The results show the feasibility of training accurate encrypted traffic classifiers with the federated learning paradigm, and the advantage of the proposed approach.

## CRediT authorship contribution statement

**Zhiping Jin:** Writing – review & editing, Methodology, Funding acquisition, Conceptualization. **Ke Duan:** Writing – review & editing, Validation, Supervision, Project administration, Funding acquisition. **Changhui Chen:** Writing – review & editing, Validation, Project administration, Investigation, Funding acquisition. **Meirong He:** Writing – original draft, Visualization, Methodology, Investigation, Formal analysis, Data curation, Conceptualization. **Shan Jiang:** Writing – review & editing, Visualization, Validation, Software, Investigation. **Hanxiao Xue:** Writing – original draft, Visualization, Validation, Resources, Methodology, Investigation, Conceptualization.

## Declaration of Competing Interest

The authors declare the following financial interests/personal relationships which may be considered as potential competing interests: Zhiping Jin, Ke Duan reports financial support was provided by Zhongshan Public Welfare Science and Technology Research Project (No. 2021B2068, 2021B2064). Changhui Chen reports financial support was provided by Science and Technology Project of Guangzhou (202102080252). Changhui Chen reports financial support was provided by Guangdong University Featured Innovation Program Project (2021KTSCX261). Zhiping Jin reports financial support was provided by Zhongshan Polytechnic Research Project (No. KYA2301). If there are other authors, they declare that they have no known competing financial interests or personal relationships that could have appeared to influence the work reported in this paper.

## Data Availability

The ISCXVPN2016 dataset used in this study is publicly available [30].
